# Cultural competence in working with the Arab Australian community: a conceptual review and the experience of the Arab Council Australia (ACA) gambling help counselling service

**DOI:** 10.1186/s40405-017-0029-0

**Published:** 2017-12-11

**Authors:** Randa Mazbouh-Moussa, Keis Ohtsuka

**Affiliations:** 1 Scorpius Antares Consulting, West Pennant Hills, NSW Australia; 2 Victoria University, Melbourne, Australia

**Keywords:** Cultural competence, Gambling counselling, Arab Australian community, Culturally and linguistically diverse (CALD) gambling

## Abstract

Although Culturally And Linguistically Diverse (CALD) communities participate less in gambling than the general population, those who gamble are more likely to show signs of disordered gambling (Moore and Ohtsuka International Gambling Studies, 1, 87–101, 2001; Raylu and Oei Clinical Psychology Review, 23, 1087–1114, 2004; Yamine and Thomas The impact of gaming on specific cultural groups, Victorian Casino and Gaming Authority, Melbourne, 2000). Research data on gambling problems and interventions in the Arab Australian community are extremely scarce. Therefore, this article will present an overview of the Arab Australian community and cultural issues regarding gambling within the Arab Australian community. Identifying these issues is important to work effectively with Arab Australians clients and those from other CALD backgrounds. The article also presents a conceptual review of peer-reviewed research articles on cultural competence in working with the Arab clients, the overview of Arab migration history to Australia and a summary of recent events that suggest a tension between Arab and non-Arab Australian communities. Observations and experiences that were encountered during the gambling counselling service operating in the Australian Arab community in New South Wales are also discussed. The research data to validate the effectiveness and positive impact of cultural competence are still in its early stages. However, a small number of community education resources have been available for working with the Arab community. From the data in annual reviews on the Arab Council Australia gambling counselling service, it was identified that cultural beliefs and expectations influence risk-taking decisions, identification of gambling issues, and preference of help seeking within the client’s social network. Further, culturally-specific sensitive issues related to political and global security events, which in turn influenced openness and willingness for the help-seeking of the Arab Australians, were identified. In conclusion, we propose that recognising diversity within the Arab Australian community is a prerequisite for increasing cultural competence and cultural sensitivity for helping professionals working with Arab Australians.

Gamblers in culturally and linguistically diverse (CALD) communities are found to be at-risk of developing gambling disorders. Yamine and Thomas (2000) reported higher prevalence rates of problem gambling in CALD communities such as Chinese, Vietnamese, Arabic, and Greek-Australians. Although CALD communities participate less in gambling than the general population, those who gamble are more likely to show signs of disordered gambling (Moore and Ohtsuka 2001; Raylu and Oei 2004; Yamine and Thomas 2000). CALD gambling research literature, although still in the early stage of development, suggests several risk factors relevant to CALD communities.

*Gambling culture and easy access* Many people from CALD communities came from countries and regions where commercial gambling is either not widely available or it is restricted. Since Australia, as well as other Western countries, has an open, permissive gambling culture (Moore and Ohtsuka 1997, 2001; Dickins and Thomas 2016; Nekich and Ohtsuka 2016), new Australians from CALD backgrounds will gain easy access to commercial gambling. Further, participation in gambling may be regarded as a milestone of acculturation to Australian culture or a “rite of passage” to adulthood (Moore and Ohtsuka 1997, 2001; Ohtsuka 2013).

*Welcoming environment* Casinos and gambling venues are welcoming entertainment venues, which provide inclusive quality customer service to all patrons including newcomers. CALD patrons, who may have limited English communication skills, are treated with respect and enjoy the gaming experience (O’Mahony and Ohtsuka 2015; Ohtsuka 2013).

*Beliefs about luck and chance* Although wishing for good fortune and good health is universal, beliefs about luck, winning and chance are influenced by culture. For example, Chinese gamblers believe in a cyclical change of luck, that ebbs and flows and is independent of human action. This belief may contribute to their higher risk-taking (Papineau 2005). Vietnamese-Australian gamblers explain luck and winning in gambling as karma; previous good deeds to others would bring gambling wins; whereas, bad deeds would bring bad luck (Ohtsuka and Ohtsuka 2010). There is also evidence that some beliefs about luck and chance are universal. For example, a belief that hard work would bring desirable outcomes or a belief in a “just world”, that bad luck in the past would be rewarded with a big win someday, is found across cultures (Ohtsuka 2013).

*Adjustment Stress and Trauma* Research literature also suggests that post-migration adjustment stress is associated with significant stress, which may trigger maladaptive coping for some new migrants (Tseng 2001). While the majority of new Australian migrants successfully make this transition, with determination and through hard work, this transition is not without stress. Some first-generation migrants may experience a considerable degree of downshifting of the vocational attainment due to their lack of English language skills, red tape preventing the recognition of their qualifications and education certificates earned in the country of origin, and obligation to support extended family members while establishing a life in a new host country. Since Australia is one of the most individualistic cultures in the world (Scott et al. 2004), new Australians from collectivist cultures may discover that their children, the second-generation Australians, reject a traditional patriarchal family structure in favour of a more democratic one. These changes during the adjustment may substantially increase the stress of the new Australians. Furthermore, some CALD migrants who arrived as refugees may have experienced traumatic events during their resettlement in Australia such as a war, living in refugee camps, physical tortures, rape and exploitation, which could result in Post-Traumatic Stress Disorder (PTSD).

It is important to recognise that adjustment stress is not limited to the first-generation immigrants but can also be substantial for the second-generation Australians. Growing up in Australia, the second-generation immigrants are more acculturated than their parents, but they have learned to live in two cultures. As a consequence, they have to negotiate between two cultural norms and may also be asked to assist older family members as interpreters. These family-related obligations could be time-consuming and cause stress.

*Reluctance to seek help* Many migrants from CALD backgrounds have limited knowledge of mental health issues, the importance of help seeking or the availability of community services. This lack of knowledge presents a significant barrier for help seeking. Likewise, cultural beliefs regarding family honour and a reluctance to consult with psychologists and counsellors, when combined with the distrust in the authorities and the establishment, further aggravate addressing the issues of addiction and confound harm minimisation efforts in the community (Yakushko et al. 2008).

## Arab Australians: who they are and where they came from

Australia is one of the most diverse countries in the world. According to the 2016 Australian Census of population and housing, more than a quarter of Australians (6163,667 people) were born overseas (26%) (Australian Bureau of Statistics 2017). Australians with an Arabic cultural background constitute 1.4% of the Australian population (321,728 people); the Arabic language is the second most frequently spoken language other than English at home in Australia (Australian Bureau of Statistics 2017). Although Arab Australians live in most Australian states and territories, the largest numbers of the Arabic speaking Australians live in Greater Sydney (194,049 people; 4.0% of the residents) followed by Greater Melbourne (76,266 people; 1.7% of the residents).

Arab Australian migration started as early as the 1880’s when the first wave of Lebanese settlers arrived in Australia. Many worked in textile, drapery and haberdashery businesses. While Syrians were the earliest Arabic speaking migrants in Australia, currently the Lebanese remain the largest and most dominant group. According to the 2016 Census, 24% of Arabic speakers (78,653) were born in Lebanon. The second largest group came from Iraq (67,352), followed by those who were born in Egypt (39,779) (Ryzk 2017).

More Lebanese migrated to Australia for economic reasons in the period following World War II, and the last wave of migration occurred at the beginning of the Lebanese civil war (1975–1990). As the civil war ended, a significant reduction of Lebanese immigration to Australia occurred. There was also an increase of Lebanese Australians wishing to return to Lebanon due to unemployment and economic downturns in Australia during the seventies (Batrouney 2001). The diversity within the Lebanese community in Australia increased following the end of the Lebanese Civil War. For instance, smaller and more diverse community associations including churches and mosques started to provide the social services to the communities and host cultural events and religious activities. However, the persistently high unemployment among the Lebanese-born population and the higher rate of unemployment in the suburbs with a higher concentration of Lebanese Australians led to an increasing of family violence, intergenerational conflicts and poverty. Problem gambling emerged as a byproduct of these social problems. (Batrouney 2000).

## Diversity within Arab culture

Diversity within the Arabic culture is often overlooked. While the term “Arabs” does not refer to a particular race or an ethnic group, the majority of Arabs are categorised as Semites; although Arabs include Caucasians, Africans, and Middle Easterners, with ancestral origins in Europe, North Africa and the Middle East. In a broad sense, it is a cultural group of multiethnic origin of peoples from the Middle East that are united by the Arabic language, and the Arabic culture.

From Oman on the coast of Arabian Sea to Mauritania in the West Africa, 22 Arabic speaking countries of the Arab league extend in a vast geographic area known as the Middle East and North Africa (MENA), which enrich the Arab culture with diverse ethnic groups and cultural traditions. Further, although the predominant religion in the Arab league is Islam, Arabic speakers include followers of *three* Abrahamic world religions, Judaism, Christianity, and Islam, which share scriptures, prophets, and cultural practices to some extent.

Arabic, widely spoken in MENA region and the official language of 22 nations in the Arab league, unites diverse Arab peoples across differences in ethnicity and religion within the Arab culture. More precisely speaking, native Arabic speakers learn regional Arabic dialects (Amiyya) from their mother or their family; then they learn the *literary* language, the Modern Standard Arabic (MSA)^1^ at school (Al-Sobh et al. 2015). MSA is the formal language of education, news media and publishing in the Arab world (Alshamrani 2012). For many Arabic speakers, MSA is a second language that they started learning at school. A parallel use of two language forms, Diglossia (Saville-Troike 1982; Trudgill 1995), makes it more complex to define the Arabic culture by shared linguistic heritage. For example, Arabic speakers in Levantine and Syria may encounter significant difficulty in understanding Maghreb (Western) Arabic dialects spoken in Morocco, Algeria, and Tunisia.

Recognising the diversity within the Arab culture is very important for understanding the Arabic culture and cultural competence to work with Arab Australians. After 9/11 attacks, the Arab communities in the West, including that in Australia, have been under scrutiny. The stereotypical view of Arabs as a single ethnic and religious group^2^ with “distinct” racial features (Naber 2000) is detrimental to gaining the trust of the Arabic community.

## Recent events affect the Arab community

The Arab communities in Western countries were affected by the political backlash after 9/11 and the counter-terrorism measures, and government surveillance. For example, Arab Americans started to question the US Census classification as white and to be recognised separately as Arabs (Zurrugh 2016). In short, Arabs in the USA are white without white “privilege” (Amer and Awad 2016; Takruri 2016). Ironically, the classification as white was originally sought to avoid the restrictions imposed by the Chinese Exclusion Act of 1882. The benefit of reclassification, however, was short-lived. The US Immigration Act of 1924 restricted Arab immigration to the United States. Arab Australians also faced similar restrictions in Australia. From the late 19th Century, the White Australia policy excluded Arabs who lived in South West Asia because they were “Asians.” Under Ottoman rule, Arabs were enemy aliens in WWI. In WWII, Arabs lived in Vichy French colonies (Lebanon, Syria, Algeria, Morocco) and Italian colonies or occupied territories (Libya, Somalia, Djibouti, Tunisia, and Western Egypt) were enemy aliens. Although gaining full acceptance after the White Australia Policy had been gradually dismantled from the 1960s, Arab Australian communities still endure the negative image of the prolonged history of war and conflict in the Middle East, the Arab–Israel conflict, the occupation of Palestine, Gaza strip and intifada. In Australia, after 9/11 attacks, the Arab Australian community experienced an increase of the racial vilifications targeting people with a Middle Eastern outlook.

The Cronulla riot is an example of how the volatile inter-community tension involving Arab Australians could escalate. On 11 December 2005, “racial” tensions turned to violence in Cronulla, one of Sydney’s beaches, after some disputes between beach users. Thousands of angry people flocked to the Cronulla beach to protest and turn Middle Easterners away from what they called “our beach” (AAP, 11th December 2005). Although seen as an intergroup conflict between the Muslim versus the non-Muslim beach users, another layer of inter-group conflicts between Pro-Riot and Anti-Riot group aggravated the riots and their aftermath (Bliuc et al. 2012). While news media may capitalise on sensationalism and focus on extraordinary events, the initial event that cascaded into inter-group conflict—that Middle Eastern beach users allegedly attacked Australian surfguards (the Australian cultural icon)—is worrisome as it exemplifies the “us versus them” dichotomy.

When a CALD community feels the surveillance and scrutiny are directed towards them, the communication and the interaction between the CALD community members and other communities will become difficult due to lack of mutual trust (O’Connor and Jahan 2014; Peek 2003). In Australia, the centre–right coalition government (under Prime Ministers Tony Abbott then Malcolm Turnbull) sought to repeal Section 18c of the Human Rights Act, but the move was defeated in 2017 (Jackson 2015; McGhee 2017). In November 2016, the Minister for Immigration, Dutton, criticised the Fraser government’s policy to accept Muslim Lebanese refugees as a mistake (Anderson 2016). The politics of fear of Islamic terrorism implicates Muslims and Arabs for complicity, making Arab Australians feel that the government policy sanctions vilification of Arabs and Muslims (Australian Arabic Council 2001). This perception would be detrimental and some Arabs Australians may feel that their path for integration is blocked (see Khawaja 2016). Berry’s acculturation studies (1997, 2005) report better mental and physical health for integration and assimilation acculturation strategies for migrants. Therefore, more explicit care should be taken to ensure that mutual trust between Arab Australians and non-Arab Australians would overcome transient issues arising from the political climate.

## Cultural competence

In the recent years and due to increased changes in cultural demographics in Australia, the counselling and clinical services approach to working effectively with clients from various cultural backgrounds slowly started to shift focus towards cultural competence. The concept of cultural competence originated in the 1980s as a way to address problems arising from inter-ethnic and cultural conflicts. Pedersen (1988) proposed a three-stage model of multicultural development for counsellors. He advocated role-play based training and simulations for counsellors to identify and overcome culturally learned stereotypes. He has also identified four dimensions of multicultural skill training and a way to develop a multicultural identity, as well as a method of applying behavioural modification techniques to effect these changes. Cultural competence was first accepted in the context of counsellor and psychologist training, to enable professionals to deal more effectively with a diverse client base (e.g., Sue and Sue 2013). Further, cultural competence was also incorporated as a requirement of personnel training in other health services such as nursing^3^ (e.g., Anderson et al. 2007; Brink 1976; Campinha-Bacote 2002) and the gaming industry personnel (Ohtsuka and O’Mahony 2012; O’Mahony and Ohtsuka 2015). Ohtsuka and O’Mahony (2012) and O’Mahony and Ohtsuka (2015) recommend that the professional staff training (both the induction and refresher training) in the gaming industry, as well as the code of practice, could include fostering employee readiness for empathy and understanding of gaming customers’ needs taking into account of their cultural point of view.

Subsequently, the notion of cultural competence was developed in the context of personnel training in health services provision and has gained currency in the formulation of government and non-government organisational policies (e.g., Australian Government National Health and Medical Research Council 2006).

Definitions of cultural competence vary greatly, from one area to another or even within a specific area, reflecting a wide range of sectors that contributed to the development of this conceptual framework. The underlying assumption of cultural competence, however, always includes the notion that it forms part of the human rights of clients who are entitled to the access to health services. That is, the health service, such as community health care and government services, should be readily available and accessible to diverse community members in a culturally sensitive manner. This requirement to account for a diverse community has become more and more important in recent years due to globalisation, and mass migration across traditional national boundaries in the 1990s to present. The concept of cultural competence, although not widely applied to a private sector such as the gaming industry, would be a useful theoretical framework to evaluate service provision, especially about harm minimisation.

In a nutshell, cultural competence is “the ability to understand, appreciate and interact with persons from cultures and belief systems *other than one’s own* based on various factors” (Medical Dictionary, n.d.). Research shows that cultural competence improves the effective prevention of chronic disease (Henderson et al. 2011), enhances the treatment of addictions (Guerrero et al. 2012) in the CALD communities (Gainsbury 2016), and that the process of care characteristics during the clinical encounter influences patients’ perceptions of clinicians’ cultural competency and affects functional outcomes (Michalopoulos et al. 2014).

Recent research on developing cultural competence in health care settings has recommended that similar culturally appropriate modification practices directed to the Arab clients are needed (Michalopoulou et al. 2014; Pediatric Nursing 2002).

Cultural competence plays a crucial factor in the provision of health services to diverse communities and clients from CALD backgrounds (Gainsbury 2016; Henderson et al. 2011). The literature search on cultural competence regarding services specifically directed to Arab Australians is scarce (Amer, 2016; Gow, n.d.). In a US study on cultural competence of counsellors, a sample of US counsellors rated their competence and confidence in working with diverse groups of clients. The findings showed that the highest percentage of the respondents reported that they lacked cultural competence to work with Arab American clients **(**Sabbah et al. 2009 **)**. Most American online cultural competence training programs focus on Arabs and Muslim Americans (The United States Department of Justice 2015a, b). While the quality of information and the availability of cultural competence training videos are commendable, it must be recognised that Arab cultural competence and Muslim cultural competence are not necessarily identical (Amer 2016). Therefore, it is likely that the difficulty of “identifying” Arab clients by US counsellors may contribute to their lack of self-reported confidence in dealing with Arab clients.

To further complicate the matter, the gambling counselling services encounter significant levels of resistance from the clients they try to help. The stigma associated with mental health and gambling, especially significant among clients from Islamic backgrounds, makes it a taboo to discuss any issues with a third-party or “outsiders” (Hamid and Furnham 2013; Youssef and Deane 2006). Participants in gambling avoid being seen or known to gamble, which is almost a universal trait of all gamblers even those from cultures where gambling is widely practised (e.g., Ohtsuka 2013; Ohtsuka and Ohtsuka 2010). In the Arabic culture, this worldview of gambling as a shameful activity impacts negatively on the Arab clients and prohibits them from talking about their gambling openly to family members, friends, and healthcare providers such as counsellors and psychologists; therefore discouraging help seeking.

In contrast, gambling is seen as an acceptable leisure activity in the Western world and the Asian countries (Binde 2005; Ohtsuka 2013; Ohtsuka and Ohtsuka 2010). Gambling fulfills entertainment needs of families or within peer groups. For example, with male friend groups seeking thrills and excitement through engagement in gambling entertainment or financial gains in sports betting (Nekich and Ohtsuka 2016); while a group of female friends may seek casual social outings to glamorous gaming venues like casinos (Ohtsuka 2013) or local pubs (Nekich and Ohtsuka 2016). A wide exposure and acceptance of gambling as a leisure activity and permissible attitudes towards gambling may be risk factors for increased cases of gambling disorders, however a permissive attitude may facilitate help-seeking behaviour as the extent of the stigma attached to gambling (or gambling disorders) is somewhat modest. Comparatively speaking, the self-disclosure of gambling-related issues is extremely difficult for the Arab clients who risk being ostracised from family, friends, colleagues, and their community.

The strong sense of extended family in the Arab culture also makes it difficult to discuss problems with outsiders. In addition, mental health issues carry high levels of stigma because mental illnesses are regarded God’s retributions or punishments. Thus, there is a high level of reluctance to seeking help from mental health professionals outside the family.

## Experiences of gambling counselling service provisions: a case of Arab Council Australia (ACA)

One of the gambling counselling treatment and recovery services, Arab Council Australia (ACA), is an ethnic specific community welfare organisation that has been providing culturally sensitive and culturally appropriate gambling counselling and related financial counselling services to the Arabic community in the Greater Sydney area since 1999 (Arab Council Australia 2016). In Melbourne, the Gambler’s Help Northern started the Arabic language gambling counselling services in 1999 (Habib 2000). The service commenced as a result of an identified need by ACA and the funding body to establish a culture-specific counselling services to the CALD communities such as the Arabic, Italian, Greek, Vietnamese and Chinese. Bilingual counsellors were employed to establish and provide the service and extensive marketing and promotional work ensured that the service was known and available to Arabic clients.

*History of the service establishment* Although the new service initially attracted low numbers of clients in South West Sydney, client numbers increased over time on a systematic trend. The following statistics indicated the effectiveness of the culturally specific and competent service provision:

The number of clients increased from 53 in 1999–1200 clients in 2016, including 2700 counselling sessions for individuals and their families. In 2013, there was funding available to expand the service into outreach regions of the Western Sydney and Coastal Sydney areas. The number of clients in these two new regions reached 121 clients and will increase based on past service statistics.

In the early stages of the service, counsellors researched the gambling behaviours of migrants in general and consulted with experts in the field of gambling addiction. As a result, it was then reported by the service that social beliefs, values and perceptions of counselling continued to be barriers to access for some clients. As has been identified in the ACA research and within the context of problem gambling counselling, formal counselling is not a common form of assistance in the Arabic speaking community due to the stigma related to gambling and associated mental health issues. The service was established based on strong foundations of research on the best available counselling therapeutic approaches and clients recruitment, maintenance and retention.

*Barriers to help seeking* Some of the main barriers and difficulties that impacted on the Arabic clients seeking help from the service were global security events. The harassment and racial vilification that the Arabic speaking community faced following the media coverage of local crimes and 9/11 attacks in 2001 impacted on a client’s mobility and motivation to attend face-to-face counselling. These barriers were addressed by the further promotion of the service by the counsellors and staff members who conducted media interviews with ethnic radios and newspapers. The service also arranged to have introductory meetings about the service with community leaders and members and with mainstream agencies.

In addition, some people from the Arab culture who participate in gambling avoid being seen or known to gamble, which is documented in the counselling records at the ACA gambling counselling service. The data indicated that clients who wished to remain anonymous and not seek help created additional barriers as effective assistance became limited. The worldview held by the Arabs that gambling is a shameful activity has negative impacts and prohibits them from talking about their gambling openly to health care providers such as counsellors and psychologists. However, it is perhaps unrealistic that cultural and religious prohibition would work as strong protective factors especially for people with Arab heritage in Western societies (e.g., Ahmed et al. 2014).

Cultural issues and norms of the Arabic communities in Australia impact on their decisions regarding seeking help. For example, collectivistic cultures in which the Arabic culture is part of have historically focused on involving the family in addressing social and behavioural issues of its members. Due to the stigma and shame attached to gambling behaviour, which is not an accepted leisure activity in the Arab world and a prohibited behaviour in Islam, some gamblers may prefer to deal with their problems with close family members or close friends (Dickins and Thomas 2016). Counselling may also be considered a foreign concept to the majority of Arab Australians especially new migrants, which leads some gamblers to address their gambling addictions and problems by talking to family members of higher status or with religious leaders (A Guide for Counsellors 2010; Youssef and Deane 2006).

To reduce the reluctance to seek help, counsellors needed a solid understanding of the Arab client’s worldview of gambling and their restricted, negative views on seeking help through counselling. Counsellors strived to work closely with clients to build trust and rapport and in doing this they aimed for good client retention rates and efficiency of treatment.

*Religious view on gambling and saving face* There has been and continues to be an appreciation in the counselling service at ACA about the significance of religion in the lives of people from the Arabic world, and of the different religious background of gambling clients. Further, counsellors are aware of the culturally sensitive issues surrounding the gambling activity within the gambler’s social network. This includes the importance of keeping one’s name and reputation in a high regard within their family and immediate community. These attitudes are connected to the deep religious and cultural beliefs, which form part of the rich history and accepted norms of the people of the Arab world and which indicate that there is shame and unacceptance about the concept of gambling where monies gained are seen as bad money not to be spent on family or health.

According to Quran (Surah Al-Baqarah [2:219]),

“They ask you about intoxicants and games of chance. Say, in them there is a great sin and [yet, some] benefits for people, But their sin is greater than their benefit.” And they ask you what they should spend. Say, “The excess [beyond needs].” Thus Allah makes clear to you the verses [of revelation] that you might give thought.

(The Noble Quran, n.d.)

In this verse, Muslims are reasoned to weigh the cost and benefit of the consequences of gambling. Gamblers are bound to become hostile to each other due to feelings of vengeance against winners and [yet,] they eventually neglect their responsibilities towards looking after their families. Gambling is not viewed as an entertainment but as a waste of time and money in which the gambler is a lazy person seeking quick monetary gains and may be involved in crime, fraud and drugs as well as being an irresponsible member of the community. Any person who gambles will be labelled as a “gambler”, and this term is associated with concepts of shame, guilt and stigma (Dickins and Thomas 2016).

*Gambling and comorbidity issue*s Many gamblers counselled at ACA presented with comorbidities of mental health issues specific to migrants and refugees, such as Post Traumatic and Stress Disorder (PTSD) (American Psychiatric Association 2013) and migration stress. These clients were subjected to threats of prosecution, to witnessing the deaths of close family members or had lived stressful war experiences and internal and external migrations before they finally settled in Australia. Counsellors and practitioners at ACA are specially trained to assist people with gambling addictions and who also require trauma treatments and interventions. Arab women, in particular, may have experienced extreme violence such as rape and torture during the war (Hamdan 2009) as well as sectarian violence in the country of origin and refugee camps.

To assist these clients and prevent additional stressors, counsellors use evidence based therapy treatments and harm reduction methods and adapt them to the client’s case and experiences. Slight changes in approaches were made to be more culturally suitable for these clients taking into account the cultural and subcultural diversity of this target group. The rationale behind modified therapeutic tools is to increase access and maintain equity principles of the service and to meet funding and accreditation requirements. In practice, counsellors translate into the Arabic language the risk and gambling behaviours assessment tools, and make changes in the contents of assessment such as omitting questions or statements which contain components of behaviour that are rarely part of the culture of the client.

*Superstitious beliefs* Another factor within the cultural beliefs of the Arabic client with gambling addictions, and which impacts on the initiation, maintenance and the likelihood of the increase of gambling activity is the use of assistive superstitious elements and rituals leading to risk-taking behaviours (Moussa 2015; Mazbouh-Moussa and Ohtsuka 2016). For example, some clients reported avoiding gaming venues on a Wednesday due to this day being a jinx. Other clients reported carrying a *hamsa* to bring luck and to protect against the evil eye (Arab Council Australia 2016). Explanations for this risk-taking behaviour may stem from the concept that these rituals and superstitious beliefs provide imaginary secondary control facing uncontrollable situations (Gmelch 2006; Malinowski 1954).

*Somatisation manifestations* Arab clients tend to use somatisation as a mode of symptom expression (cf. psychologisation) and regard affective disorders as caused by somatic origins (AL-Krenawi and Graham 2000; Hamdan 2009). Somatisation as a mode of symptom expression is also reported for other migrant groups such as Vietnamese Australians (Ohtsuka 2005). Therefore, counsellors need to interpret physical symptoms in medical conditions that may represent somatic expressions of psychological origin such as stress and anxiety. Physical symptoms of a number of ACA clients, both men and women, were signs of somatisation such as back pain, chronic tiredness and lethargy. Research suggests that the mental health needs for Arab women who seek counselling are similar to that of women from other cultures, and women, compared to men, show a higher prevalence of depression and anxiety (Hamdan 2009). Depression and anxiety were commonly observed among the help-seeking Arab Australian clients. For women, it was observed that loneliness, marital and relationship problems were also related to gambling disorders.

*Client engagement strategies* To engage with the client and to discuss their concerns about counselling, practitioners at ACA decided to involve close members of the gambler’s family in both the treatment and recovery processes. While this approach does not align fully with the Western principle of protecting the client’s privacy, it does lead to longer contact and engagement with the client. This is supported by research conducted by the Centre for Gambling Research which showed that members of ethnic cultural groups in Canberra who were facing gambling addictions turned most often to families and friends, or to other generic agencies for help (McMillen et al. 2004).

*Marketing campaigns* Marketing campaigns about the availability of gambling counselling are based on culturally appropriate designs and ease of access to all community members. Some of the media and marketing activities that were developed by the service in partnership with the funding body and a larger multicultural gambling counselling organisation were the production of video/DVD/TVC short documentaries about problem gambling and its impact on the Arabic gamblers and their families and community (Gamblers Help/NSW. 2012a, b). The production featured interviews with a prominent and respected Arabic medical General Practitioner (GP) in South West Sydney and an Arabic counsellor providing their thoughts about gambling and its multiple forms and preferences within the Arabic culture. The GP and counsellor provided their guidance to the audience about the consequences of problem gambling and enforced and normalised the behaviour of help-seeking and its benefits to the person affected and their families. Arabic speaking GPs and counsellors who deliver educational messages on social issues to community members often succeed in their mission to influence positive social change. For example, a self-identified problem gambler in Canberra quoted that “Our family doctor, who has treated our family for 25 years, has been of assistance in dealing with these problems” referring to his gambling problems, alcohol abuse and stress (McMillen et al. 2004).

Another video production was developed about the myths and facts of gaming machines and the probability of winning when playing the poker machine (Gambler’s Help/NSW 2009). A well-known actor from a Pacific Islander community was selected as a presenter in the video. The involvement of popular, prominent and trusted public figures in media to deliver messages proved to be an efficient way to reach the hearts and minds of community members, with the aim of creating initial stages of social and behavioural change. It is worth mentioning that all these videos are presented in spoken Arabic with English subtitles to ensure presentations of cultural competence and to align with the access and equity principles of the rights of community members to receive education and awareness about the concept of gambling and its implications on the person affected when it becomes a problem. The use of community languages in the service delivery and the availability of translated resources are crucial to gain trust within the community and overcome the initial resistance against seeking help from “outsiders.” For a similar reason, research publications about particular cultural and language groups should include an abstract written in the community language. This would maximise the information dissemination to the community, which is a focus of the research effort. The appendix of this article thus includes the abstract in the Arabic language.

*Observations and testimonials* Counsellors at ACA developed culturally appropriate counselling to address issues relating to the client’s resistance to seek counselling and to engage respectfully with clients. They integrated Western based therapeutic approaches (such as cognitive behavioural therapy) with the innovative culturally appropriate Eastern based therapeutic approaches to minimise the client’s stress and anxiety due to feelings of guilt and shame to promote full recovery. For example, counsellors successfully assisted one of the clients who held great importance to their religion and whose reputation was impacted enormously by what his family and community members thought of him. The counselling approach was to, during talk therapy, highlight the client’s religion and social status by discussing religious rules, prohibitions and expectations and their meanings that may lead the client to make positive behavioural changes.

The therapy focused on the status of the client within his family circle and the importance of what his children and relatives think of him as the head of the family and the noble social expectations of him. The client then had to look at both life choices and what has more value to him about his immediate social environments. This method of combining traditional talk therapy methods about one’s own beliefs and social status with the evidence based existing psychotherapy approaches yielded positive results for the client regarding achieving their goals.

## Recommendations to increase cultural competence within gambling counselling services

It is concluded that from many years of experience working with the Arabic clients, the bilingual counsellors who speak the Arabic language (both Arabic dialects and Modern Standard Arabic) and who understand the culture and the social and political background of the client can assist and provide efficient and engaging treatment and recovery services to the person who is seeking help to either abstain or decrease their unhealthy gambling behaviours (Arab Council Australia 2016).

Based on the case discussion of the ACA gambling counselling experience as an example of culturally competent client services, we conclude that more research is needed on the application of cultural competence in the Australian context and gambling counselling services working with CALD communities to develop the best practice and cultural competence measures. Further research may also create more understanding of gambling counselling service provisions to assist clients from CALD backgrounds in a more effective way. For example, culturally competent practitioners will be able to improve the process of care by understanding the clients’ worldviews (e.g., Michalopoulou et al. 2014). After all, it is not so much about whose worldview is closer to truth but more about how the practitioner understands the client’s view of the world and seeks explanations of addictive behaviours. This would ultimately improve the counselling outcome.

## Footnotes

1 MSA is derived from the Classic Standard Arabic in which Qur’an was written.

2 Arabs accounts for a minority (15%) in the total numbers of Muslims in the world (Jandt 2013).

3 However, cultural dimensions of care was mentioned by Florence Nightingale (DeSantis and Lipson 2007).
